# Particle release and control of worker exposure during laboratory-scale synthesis, handling and simulated spills of manufactured nanomaterials in fume hoods

**DOI:** 10.1007/s11051-018-4136-3

**Published:** 2018-02-21

**Authors:** Ana S. Fonseca, Eelco Kuijpers, Kirsten I. Kling, Marcus Levin, Antti J. Koivisto, Signe H. Nielsen, W. Fransman, Yijri Fedutik, Keld A. Jensen, Ismo K. Koponen

**Affiliations:** 1 National Research Centre for the Working Environment (NRCWE), Lerso Parkallé 105, 2100 Copenhagen, Denmark; 2 TNO, Risk Analysis for Products in Development, Zeist, The Netherlands; 3 PlasmaChem GmbH, Schwarzschildstr 10, 12489 Berlin, Germany

**Keywords:** Airborne nanoparticles, Nanomaterial synthesis, Nanomaterial handling, Emissions, Exposure assessment, Fume hood, Environmental health and safety issues

## Abstract

Fume hoods are one of the most common types of equipment applied to reduce the potential of particle exposure in laboratory environments. A number of previous studies have shown particle release during work with nanomaterials under fume hoods. Here, we assessed laboratory workers’ inhalation exposure during synthesis and handling of CuO, TiO_2_ and ZnO in a fume hood. In addition, we tested the capacity of a fume hood to prevent particle release to laboratory air during simulated spillage of different powders (silica fume, zirconia TZ-3Y and TiO_2_). Airborne particle concentrations were measured in near field, far field, and in the breathing zone of the worker. Handling CuO nanoparticles increased the concentration of small particles (< 58 nm) inside the fume hood (up to 1 × 10^5^ cm^−3^). Synthesis, handling and packaging of ZnO and TiO_2_ nanoparticles did not result in detectable particle release to the laboratory air. Simulated powder spills showed a systematic increase in the particle concentrations inside the fume hood with increasing amount of material and drop height. Despite powder spills were sometimes observed to eject into the laboratory room, the spill events were rarely associated with notable release of particles from the fume hood. Overall, this study shows that a fume hood generally offers sufficient exposure control during synthesis and handling of nanomaterials. An appropriate fume hood with adequate sash height and face velocity prevents 98.3% of particles release into the surrounding environment. Care should still be made to consider spills and high cleanliness to prevent exposure via resuspension and inadvertent exposure by secondary routes.

**Electronic supplementary material:**

The online version of this article (10.1007/s11051-018-4136-3) contains supplementary material, which is available to authorized users.

## Introduction

Manufactured nanomaterials (NMs), which in European regulation currently are considered particulate materials in any shape where by number 50% of the objects have at least one dimension between 1 and 100 nm (COM 2011), are important constituents in current global technological developments (Savolainen et al. 2013; ISO 2015). Their importance is mainly ascribed to the fact that they simply due to the nanoscale often possess enhanced or even new properties as compared with their bulk material counterparts and not least that it today is possible to design their chemical and structural characteristics at the atomic to a nanometre scale (Feynman 1960). However, development, production and industrial use of these new or only partially known materials result in potentially new emerging risks. Besides possible change in toxicological effects, the nanosize of the primary particles is associated with a risk of exposure to very small particles that have considerably higher deposition efficiencies in the sensitive alveolar compartment of the human airways.

It is expected that the highest risk of NM exposure to humans will be at workplaces (Maynard and Pui 2007; Tsai et al. 2011; O’Shaughnessy 2013; Fonseca et al. 2015; Koivisto et al. 2012, 2017; Viitanen et al. 2017). While exposure to NMs through inhalation is considered the main uptake route, oral exposure and dermal exposure, which can result in inadvertent oral exposure are generally considered to be of secondary importance (Yi et al. 2013; Larese Filon et al. 2016). Thus, it is important to identify, characterise and assess the potential NM exposure scenarios through the entire innovation and NM product life-cycle to enable adequate risk management (Clark et al. 2012; Aitken et al. 2011), which is currently requested under the Registration, Evaluation, Authorization and Restriction of Chemicals (REACH) regulation (ECHA 2016).

Previous survey of nanosafety practices in laboratories revealed that the NM exposure risk is potentially high due to insufficient emission controls and poor use of personal protective equipment (PPE; Balas et al. 2010). During synthesis and handling of NM, 47.5% of the questionnaire respondents used standard laboratory fume hoods as engineering control (Balas et al. 2010; European Committee for Standardisation 2003). Despite usually producing and handling of low masses as compared with industrial scales, there has been evidence of relatively high risk of exposure during laboratory synthesis, handling, packing and cleaning activities (Dahm et al. 2013; Gomez et al. 2014; Demou et al. 2008, 2009; Curwin and Bertke 2011; Ding et al. 2017).

Studies on factors affecting the performance of laboratory fume hoods revealed that the height of the sash opening, hood face velocities, airflow patterns inside the hood, operator hand-arm-trunk motions and thermal conditions are the most critical parameters (Ahn et al. 2008, 2016; Johnson and Fletcher 1996; Guffey and Barnea 1994; Tsai 2013). Particular working conditions, such as working with a fully open sash, arms down at sides posture, as well as the presence of thermal sources and clutter inside the fume hood, result in the poorest hood performance and exposure at the worker’s breathing zone (Ahn et al. 2016). As part of a preventive maintenance programme, the compliance to the European Committee for Standardisation (2003) standards or equivalent protocol is a compulsory measure aimed at ensuring the safety of laboratory workers and good performance of fume hoods (EN14175). However, assessment of the performance of fume hoods under real NM handling scenarios has received little attention in the scientific literature. Lee et al. (2011) observed a noticeable increase in airborne particle number concentrations (up to 4.6 × 10^4^ cm^−3^) with a bimodal distribution (< 30 and 70–100 nm) during nano-TiO_2_ manufacturing in a fume hood. Tsai et al. (2009) also detected a significant release of NMs (reaching 7 × 10^3^ cm^−3^ above background) into the workplace air while handling and harvesting dry nanoalumina and nanosilver in a laboratory fume hood with adequate sash height (0.3–0.5 m; NIOSH 2012) and an optimum range for hood face velocity (0.5–0.6 m s^−1^) as recommended by the American Conference of Governmental Industrial Hygienists (ACGIH 2007) and the European Committee for Standardisation (2003). Face velocities below the range 0.4–0.6 m s^−1^ are insufficient to avoid influence of room air flows, and quick worker movements can result in particles release from the fume hood while face velocities above 0.4–0.6 m s^−1^ can create excessive turbulence and thereby cause release of nanoparticles out of the fume hood (Tsai et al. 2009). Additionally, special attention should be taken in case of occurrence of real accidental events such as the spillage of NMs (Gomez et al. 2014).

With the aim to complement previous studies with relevant information on the performance of fume hoods under real case studies, we studied the release and occupational exposure of dust particles during small-to-medium-scale production and handling of CuO, TiO_2_ and ZnO NMs in two different laboratories. Additionally, we studied the exposure control efficacy of a standard laboratory fume hood by simulating spillage using NMs with different dustiness indices (DI) under different drop heights and mass loads.

## Materials and methods

The measurement plan included real-time particle monitoring and collection of samples for gravimetric, morphological and semi-quantitative chemical analysis in near field (NF), far field (FF) and personal (breathing zone (BZ)) during working and non-working periods. The non-working periods were used to define the background (BG) concentrations at all measurement points (NF, FF and/or BZ) using the measurements obtained 10 to 30 min prior the target activity. The BG consisted of particles from other processes that may occur in laboratory surroundings, infiltration processes of outdoor particles in indoors and/or particles from earlier processes. This approach assumes the BG to be constant and spatial and temporal background variations were not considered (Kuhlbusch et al. 2011).

### Instruments and techniques

The online (real-time) methods employed in this study aimed to study airborne particle total number concentration and size distributions in the range from 2.5 nm to 20 μm by using the following monitoring instrumentation:

- Optical particle sizer (OPS; TSI model 3330, TSI Inc., Shoreview, MN, USA; Baron and Willeke 2001; TSI 2012; McMurry 2002) to measure the optical particle size distributions in 16 channels from 0.3 to 10 μm in 1-s interval.
- Fast-mobility particle sizer (FMPS; TSI model 3091, TSI Inc., Shoreview, MN, USA; Asbach et al. 2009) for determination of particle mobility size distributions in the range 5.6–560 nm in 32 size channels in 5-s time interval.
- Aerodynamic particle sizer (APS; TSI model 3321, TSI Inc., Shoreview, MN, USA; Peters and Leith 2003) for determination of aerodynamic particle size distribution in the range 542 nm–20 μm in 1-s time resolution.
- Portable condensation particle counter (CPC; TSI model 3007, TSI Inc., Shoreview, MN, USA; Matson et al. 2004; TSI 2007) to measure the total particle number concentration from 10 nm to > 1 μm in 1-s time resolution.
- Ultrafine water-based CPC (UWCPC; TSI model 3786; TSI Inc., Shoreview, MN, USA; Liu et al. 2006; Hering et al. 2005), to measure the total particle number concentration from 2.5 nm to > 3 μm in 1-s time interval.
- Diffusion size classifier miniature (DiSCmini, Matter Aerosol AG, Wohlen, Switzerland; Fierz et al. 2011) to measure total particle number, mean particle diameter, and the LDSA of particles in the size range of 10–700 nm with 1-s time resolution.
- Dust monitor (model 1.109, Grimm Aerosol Technik, Ainring, Germany; Peters et al. 2006) to measure the optical particle size distributions in the range 250 nm–30 μm with 1-min time resolution.

The room temperature (*T*) and relative humidity (RH) was measured by a Gemini TinyTagPlus (TGP-1500, Gemini Data Loggers Ltd., West Sussex, UK) whenever are mentioned in the text.

The offline methods employed in this study consisted in:

- Collection of respirable dust for gravimetric and inorganic chemical analysis by using Fluoropore™ membrane filters 37-mm PTFE with 0.8-μm pore size (Millipore, Billerica, MA, USA) mounted in sampling cyclones GK2.69 (BGI Inc., Waltham, MA, USA) connected to portable sampling pumps (Apex2, Casella Inc.) operating at 4.2 l min^−1^ (Stacey et al. 2014). Respirable particle mass concentrations were gravimetrically determined by pre- and post-weighing the filters collected using an electronic microbalance (Mettler Toledo Model XP6) with ± 1 μg sensitivity located in a climate controlled weighing room (RH = 50% *T* = 22 °C). Three blind filters were stored to be used as laboratory blanks to correct for handling and environmental factors. After weighing, the sampled filters were stored for subsequent inorganic chemical composition characterisation by wavelength dispersive X-ray fluorescence analyser (WDXRF Tiger S8, Bruker, Karlsruhe, Germany).
- Collection of airborne particles on 400-mesh Cu grids pre-coated with holey carbon film by using mini-particle sampler (MPS) connected to a pump (Apex2, Casella Inc.) operating at 0.3 l min^−1^ during 1-min sampling time.
- Aerosol samples collected by MPS were analysed in a transmission electron microscope (TEM; Tecnai T20G2 FEI, Eindhoven, The Netherlands) with an 80-mm^2^ silicon drift energy-dispersive spectrometer (EDS) (Oxford, UK). High-resolution images were recorded with DigitalMicrograph software (Gatan Inc., Pleasanton, VA, USA) using a bottom-mounted camera (Gatan US1000). Mineral phases were analysed by selected area diffraction patterns on agglomerates of multiple single crystals. In situ EDS chemical analysis of agglomerates and individual particles were performed with an acquisition time of 100 s.
- Collection of surface samples by using a wipe sampler device consisting of a PVC housing (6.5 × 2.5 × 2.5 cm) in which a wipe sampling head (diameter of 2.5 cm) was attached together with a spring for a continues force at the surface. The plate house retainer consisted of a PVC plate (14 × 10 × 0.5 cm) in which three tracks were grounded. The sampling device travels from one side of the track to the other, resulting in a monitored surface area of 22 cm^2^. Circular samples (diameter of 3.5 cm) were cut from a cotton glove (stretch cotton, 240 g m^−2^, v/d Wee, Riel, The Netherlands) to act as wipe medium. Prior to use, the samples were stored in a desiccator for at least 48 h.
- Surface wipe samples were analysed using a Tescan MIRA-LMH Field Emission Gun Scanning Electron Microscope (FEG-SEM) operated at an accelerating voltage of 15 kV and equipped with a Bruker AXS Energy Dispersive X-ray (EDX) spectrometer with a Quantax 800 workstation and a XFlash 4010 detector. Automated particle analysis was performed using the Scandium SIS software package (Olympus Soft Imaging Solutions GmbH, Germany).

### Synthesis and handling of NMs in an industrial research laboratory

The production of three NMs (CuO, ZnO and TiO_2_) was performed by thermal decomposition of sol-gel-synthesised inorganic precursors (Cu_2_(OH)_2_CO_3_, Zn_5_(OH)_6_(CO_3_)_2_ and TiCl_4_, respectively), washed and dried. The physicochemical characteristics as well as the morphology of the three different pristine inorganic nanostructured NMs assessed in this study are shown in Table S1 in the Electronic supplementary material. The primary produced nanoparticles are mainly polydisperse spherical and can range from a few to approximately 40 nm in mean diameter. Only the ZnO nanoparticles had a nearly monodisperse size distribution. Morphologically, the pristine NMs were mainly aggregates and agglomerates of primary nanoparticles (Table S1 in the Electronic supplementary material).

**Fig. 1:**
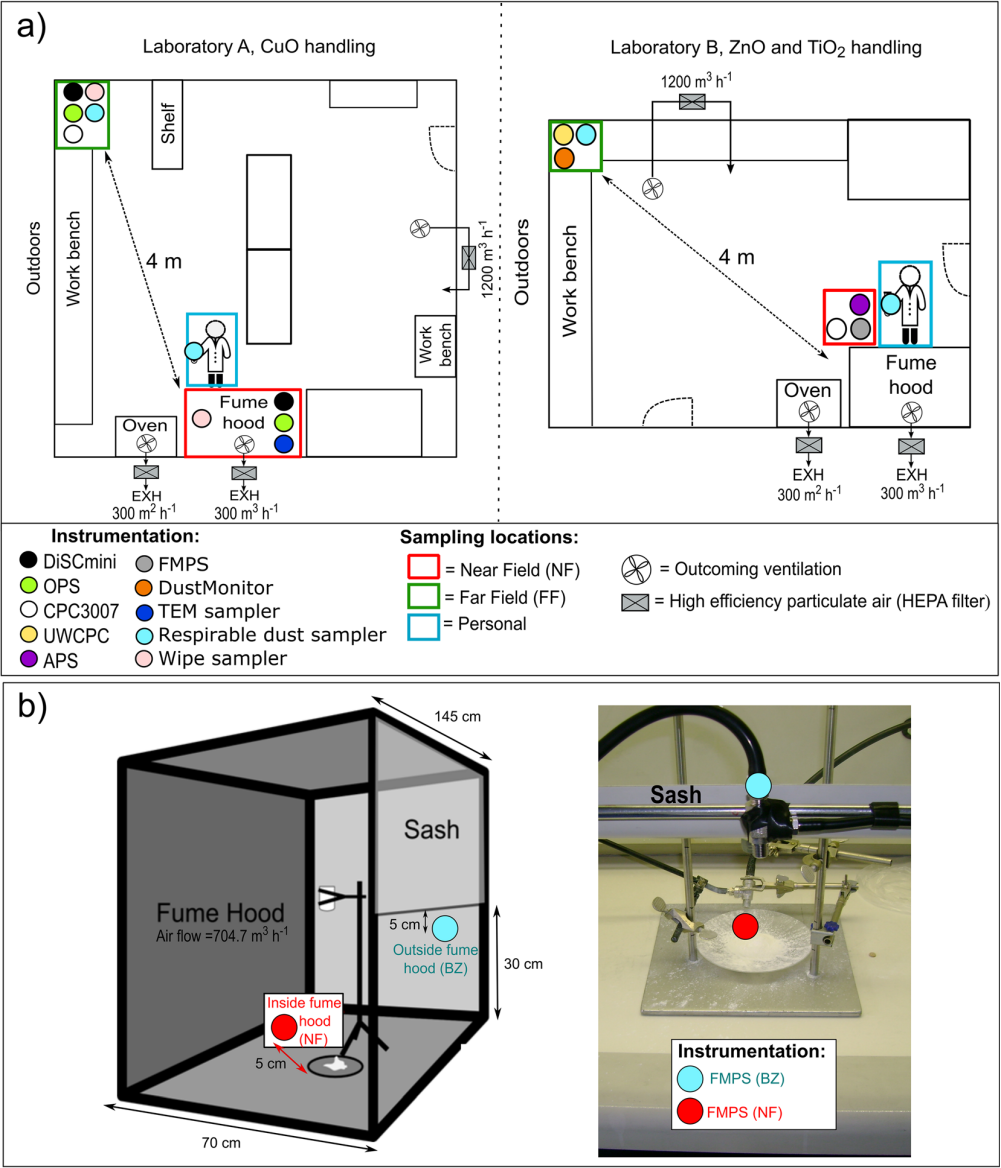
Layout of **a** the work environment in both laboratories A and B showing as red, green and blue, the near-field, far-field and personal sampling locations, respectively, and **b** drop test experiments

Field measurements were conducted in two separate laboratories, named laboratories A and B, respectively (PlasmaChem GmbH, Berlin, Germany). The layout of these two work areas and placement of the measurement devices is shown in Fig. 1a. A researcher synthesised and handled CuO NMs in Laboratory A and ZnO and TiO_2_ in laboratory B. Both laboratories were mechanically ventilated with HEPA-filtered outdoor air at a volume flow rate of 1200 m^3^ h^−1^ corresponding to air exchange ratios of 9 and 2 h^−1^, respectively. Among the processes involved in the NM synthesis and handling, four different operational activities with highest potential of particle release or secondary particle formation were identified and monitored:

1. Milling of 1 kg of solid-phase inorganic precursor under the fume hood to make the next step of calcination process more homogeneous in terms of particle formation (only done and assessed in laboratory A for CuO in the form of copper hydroxyl carbonate (Cu_2_(OH)_2_CO_3_));
2. Synthesis of NM: calcination of the inorganic precursors using a conventional oven at approximately 350 °C for approximately 2 h;
3. Transferring and natural cooling down of the produced NM (approximately 25% less than the original precursor total mass) in a polyethylene (PE) container under the hood;
4. Packing the material in glass flasks or PE bottles, both equipped with a hermetic closure, under the hood (only done and assessed for ZnO and TiO_2_ in laboratory B).

It should be noted that measurements were not made during preceding work step tasks, such as sol-gel synthesis of the precursors, sedimentation, washing and drying, where NM was not present.

The calcination of the inorganic precursors (task 2) was carried out in an oven with local exhaust ventilation (300 m^2^ h^−1^). Tasks 1, 3 and 4 were carried out either in laboratory A or B in a standard laboratory fume hood (1.35 m height, 1.8 m width and 0.7 m depth; hood type *Secuflow*, model AC2, Waldner GmbH, Germany) at a constant exhaust flow (300 m^3^ h^−1^) with half-open sash (40–50 cm; face velocity of 0.1 m s^−1^). The worker used cotton laboratory coat, safety goggles and filtering face piece respirators (type FFP3).

The parallel stationary measurements and sample collection were done from a height of 1 to 1.3 m at the NF (close to the potential emission source) and at the FF located 4 m distance from the working area (Fig. 1a). In laboratory A, the source domain measurement (NF) was under the fume hood ca. 2 to 3 cm from the source to laboratory B was at the side of the worker < 1 m from fume hood opening. Additionally, personal respirable dust and TEM and surface wipe samples were collected.

### Drop test experiments

Figure 1b illustrates the setup for the drop test experiments conducted to simulate accidental spills. This study was carried out in a small laboratory (area = 21 m^2^) with a HEPA-filtered general room-air ventilation rate of 550 m^3^ h^−1^. Containers located at a height of 5, 10, 20 or 40 cm filled with up to 125 g powder of silica fume, zirconia TZ-3Y and TiO_2_ NMs, were rapidly tipped over by a laboratory technician to simulate an NM spillage inside the fume hood. The characteristics of the used NM are described in Table 1. Different combinations of drop heights and material amounts were tested to investigate the particle emission during the drop and the emission caused by the impact of the dust to the bottom of the fume hood. At least two replicas of each drop test were performed, and in between, the contaminated surface of the fume hood was cleaned. Cleaning followed recommended procedures such as described by NIOSH (2012) and included removal of the glass Petri dish, vacuuming cleaning of surfaces using a HEPA-filtered vacuum cleaner designed for asbestos cleaning, followed by wet wiping with ethanol. The studied fume hood (Holm and Halby A/S, Denmark) operated with an exhaust flow of 704.7 m^3^ h^−1^ which passes through two exhaust ducts located at the bottom and on the top of the fume hood, respectively. The sash height was 30 cm, and the width of the opening was 145 cm, which results in an average hood face velocity of 0.45 m s^−1^. During the tests, there were no other activities or disturbances in the laboratory. A worker sat in front of the hood in a typical working position where her nose was positioned 5 cm outside the sash plane. Particle measurements were carried by using FMPS, TSI model 3091 simultaneously at two different positions (Fig. 1b). The NF position was placed inside the fume hood at a distance of 5 cm from the powder beaker, while the breathing zone (BZ) measurement position was located outside (5 cm below the sash plane) and within a 30-cm radius of the worker’s nose and mouth.

**Table 1:** Physicochemical characteristics and corresponding manufacturer of the NM under drop test experiments

Material	Manufacturer	Mean particle size (nm)	Specific surface area (m^2^ g^−1^)	Bulk density^a^ (g cm^−3^)	DI_inhalable_^a^ DI_respirable_^b^ (mg kg^−1^)	*S*_single drop_^a, e^ *S*_continuous rotation_^a, e^ (× 10^−7^ μm^3^ s^−1^)
Silica fume (microsilica grade 920; CAS No. 69012-64-2)	Elkem AS, Silicon Materials (Oslo, Norway)	150^c^ trace of quartz < 10 nm^a^	12^d^	0.26^a^	702 ± 291ª 7 ± 4 (<DL)^b^	2.6 39.4
Zirconia TZ-3Y (72% (Y,Zr)O_2_; CAS No. 308076-80-4)	TOSOH Corporation (Tokyo, Japan)	26.9 nm (X-ray diffraction) granules with diameter range 10–60 μm^a^	15.4^c^	1.5^a^	283 ± 43ª N/A	0.06 4.8
TiO_2_ (UV-Titan M111 100% rutile TiO_2_; CAS No. 13463-67-7)	Kemira Oy (now Sachtleben)	18.6 (X-ray diffraction)^a^	111^d^	0.66^a^	8338 ± 233ª 1050 ± 38^b^	17.2 264.3

N/A, not available data; <DL, below detection limit

^a^Data from Schneider and Jensen (2008)

^b^Previously unpublished data from Jensen, Nielsen and Levin produced independently from inhalation dustiness values according to the method described in Jensen et al. (2016)

^c^Data from manufacturer

^d^Data from Vippola et al. (2009)

^e^Total respirable volume emission (*S*) measured by APS

### Data analysis

For the reason of using different instrumentation, the probability value (*p* value) was calculated by the two-sample *t* test (unequal variances) so it can provide insights into the agreement between data in NF, FF and/or BZ for the BG and work activities in terms of mean particle size diameter and total particle number concentrations. If the *p* value is less than or equal to the significance level (*α*, set at 0.05), the test suggests that the observed data are inconsistent with the null hypothesis, so the sample differs significantly. In this study, a test with a *p* value ≥ 0.05 was considered to reflect that there was no difference between the data.

The fume hood efficacy (*ε*) was calculated as:

1 $$ \varepsilon\ \left(\%\right)=1-\frac{N_{\mathrm{Spill},\kern0.75em \mathrm{BZ}}-{N}_{\mathrm{BG},\kern0.75em \mathrm{BZ}}}{N_{\mathrm{Spill},\kern0.75em \mathrm{NF}}-{N}_{\mathrm{BG},\kern0.75em \mathrm{NF}}}\times 100 $$

where *N*_BG, BZ_ and *N*_BG, NF_ are the BZ and NF background particle number concentrations measured prior to the spill activity (20 min averaged) and subtracted to *N*_Spill, BZ_ and *N*_Spill, NF_ which are the BZ and NF particle number concentrations during spill, respectively.

## Results

### CuO synthesis and handling at laboratory A

**Fig. 2:**
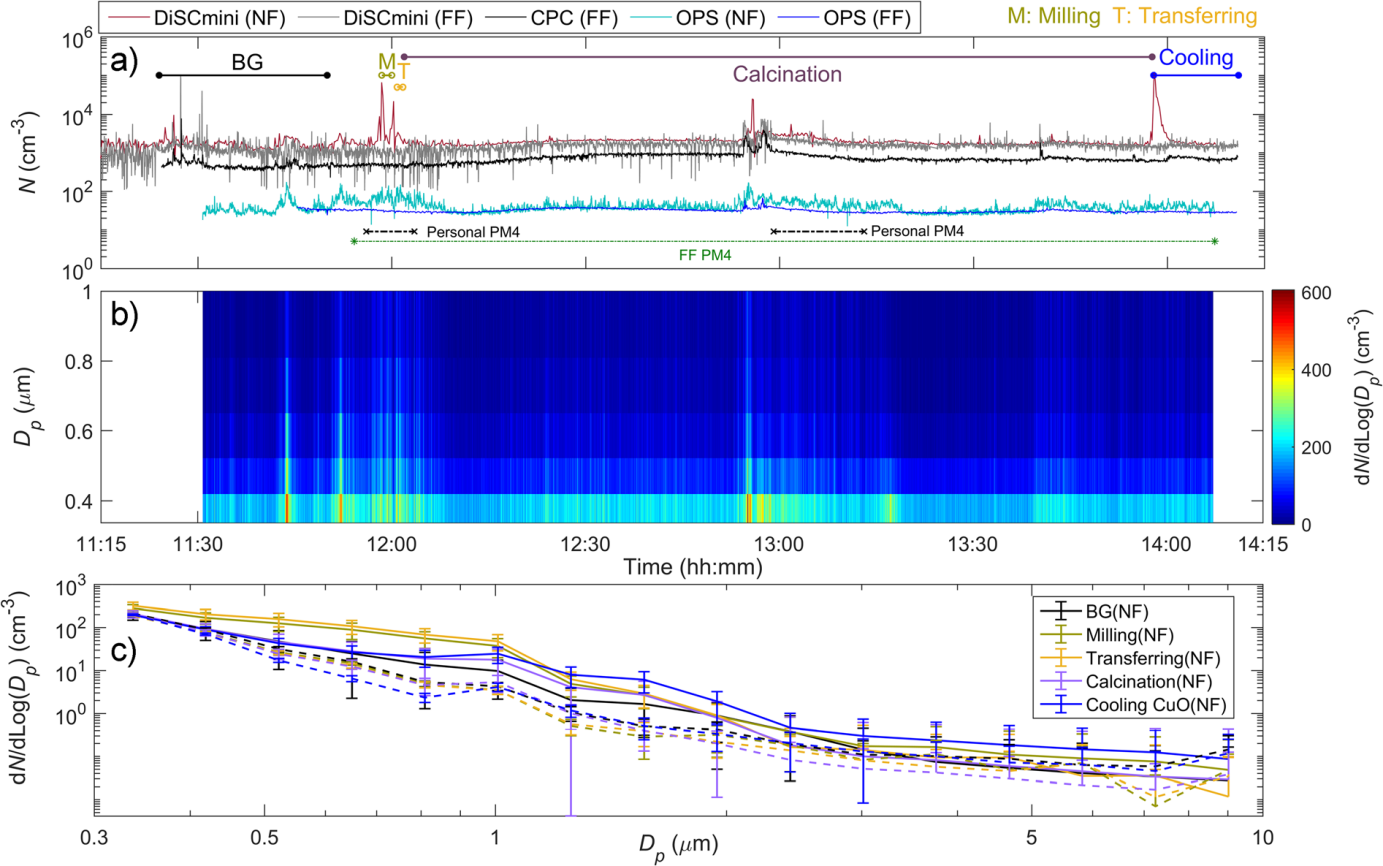
Time series of **a** total particle number concentrations measured simultaneously at NF (in the fume hood) and FF (4 m away from fume hood) during CuO synthesis and handling over 3 h period; **b** particle number size distributions obtained by OPS (range 0.3–10 μm) in the NF; and **c** mean particle size distribution measured by OPS in NF (solid lines) and FF (dashed lines) during each task. The whiskers show the standard deviation. (For interpretation of the references to colour in this figure legend, the reader is referred to the web version of this article)

**Fig. 3:**
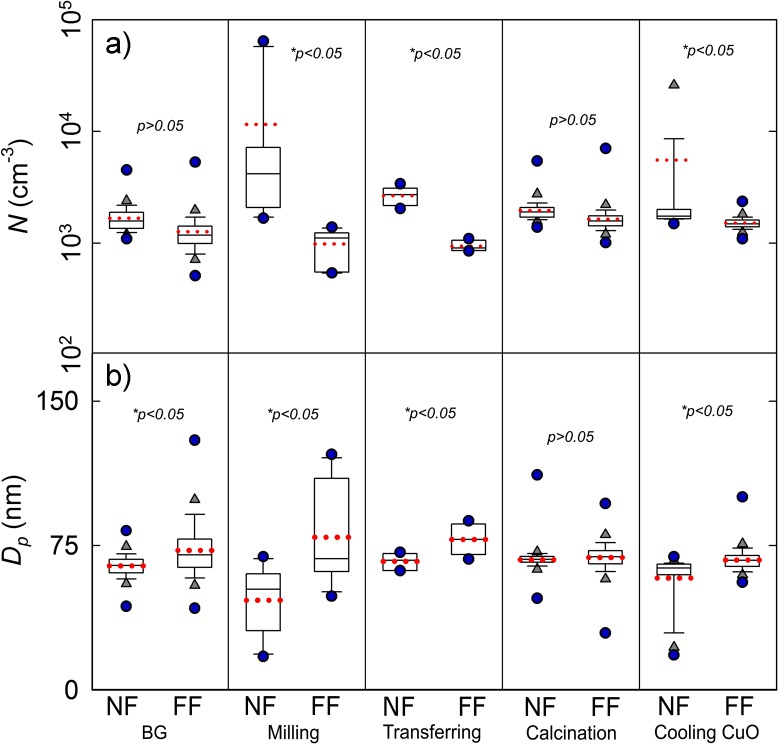
Vertical box plots for the task-specific particle NF and FF measurements: **a** particle number concentration (*N*; DiSCmini, range 10–700 nm) and **b** mean particle diameter (*D*_p_; DiSCmini, range 10–700 nm). The lower and upper limits of the box plots represent the 25th and 75th percentiles, and the line within the box marks the median. Whiskers (error bars) above and below the box indicate the 90th and 10th percentiles and the above and below grey triangles represent the 95th and 5th percentiles. In addition, the mean is shown as red dotted line and the outlying blue circles as minimum and maximum. *p < 0.05 was considered to reflect that there is a significant difference between the data

Particle measurements during synthesis, handling and packaging activities of CuO nanoparticles in laboratory A are illustrated in Figs. 2 and 3 and summarised in Table S2 in the Electronic supplementary material. The average day temperature and relative humidity measured in the laboratory were 22 ± 0.2 °C and 35 ± 0.8%, respectively.

Statistically significant short-term total particle number concentration peaks were exclusively registered by the NF DiSCmini during milling (*N*_max_ = 6.5 × 10^4^ cm^−3^), transferring (*N*_max_ = 3.4 × 10^3^ cm^−3^) and cooling of CuO (*N*_max_ = 1.2 × 10^5^ cm^−3^) and had mean diameters of 47 ± 17 nm, 67 ± 4 nm and 58 ± 13 nm, respectively (Figs. 2 and 3). These NF peak concentrations were increased from BG (mean *N* = 1.7 ± 0.4 × 10^3^ cm^−3^) by a factor of 2 to 72. In contrast, the particle concentration levels measured in the FF remained at BG levels (on the order of 10^3^ cm^−3^) with coarser mean particle diameters.

The FF airborne particles were mainly below 100 nm in diameter (Fig. 3b) and the particle concentrations measured by the OPS were below 74 cm^−3^. Low NF/FF ratios (< 2.4) measured by the OPS shows that particles coarser than 300 nm were not produced nor released during these processes to any great extent (Table S2).

Exceptionally, an increase in particle number concentrations above BG was measured during calcination process between 12:55 and 13:00 in both NF and FF (Fig. 2a). These peaks were attributed to resuspension of coarse particles upon entrance of personnel in the laboratory at 12:55.

Respirable dust concentrations were analysed from samples collected in FF during the entire work activity period (133 min; Fig. 2a) and from BZ (personal) during CuO synthesis and handling (21 min; Fig. 2a). The measured BZ and FF mass concentrations were below the minimum detection limits of 161 and 26 μg m^−3^, respectively. The WDXRF analysis revealed exclusively elements of Cl, S and Si which most likely originated from outdoor air.

**Fig. 4:**
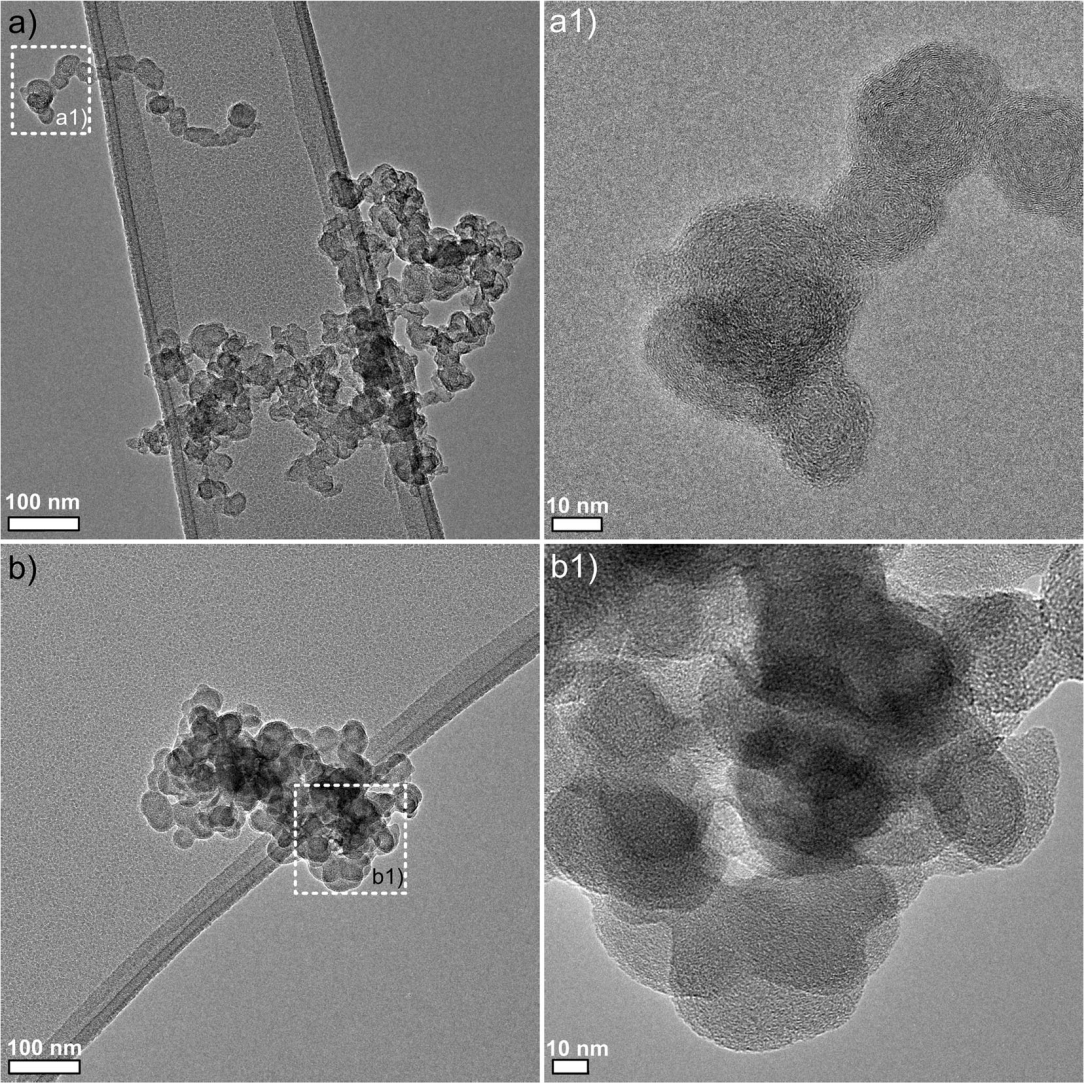
TEM images of nanoparticles collected under the fume hood during: **a** milling of Cu_2_(OH)_2_CO_3_ and **b** natural cooling of CuO NM. Corresponding zoom of the selected areas are identified in pictures a1 and b1, respectively. The fine structure resembles diffusion flame soot, as for example produced by a Bunsen burner

The TEM analyses confirmed the release of particles and risk of exposure depending on the protection efficiencies of the fume hood (Fig. 4). Though CuO nanoparticles were not observed by TEM, soot agglomerates of diffusion flame character were consistently found, during milling of the solid-phase inorganic precursor Cu_2_(OH)_2_CO_3_ (Fig. 4a) and during natural cooling down of CuO NM (Fig. 4b). These soot particles were most probably originated from the processes or another indoor source. Similarly, no Cu-based particles were observed in the SEM analysis of the sample wiped in the surface of the fume hood at the end of the synthesis and handling activities.

### ZnO and TiO_2_ synthesis and handling at laboratory B

**Fig. 5:**
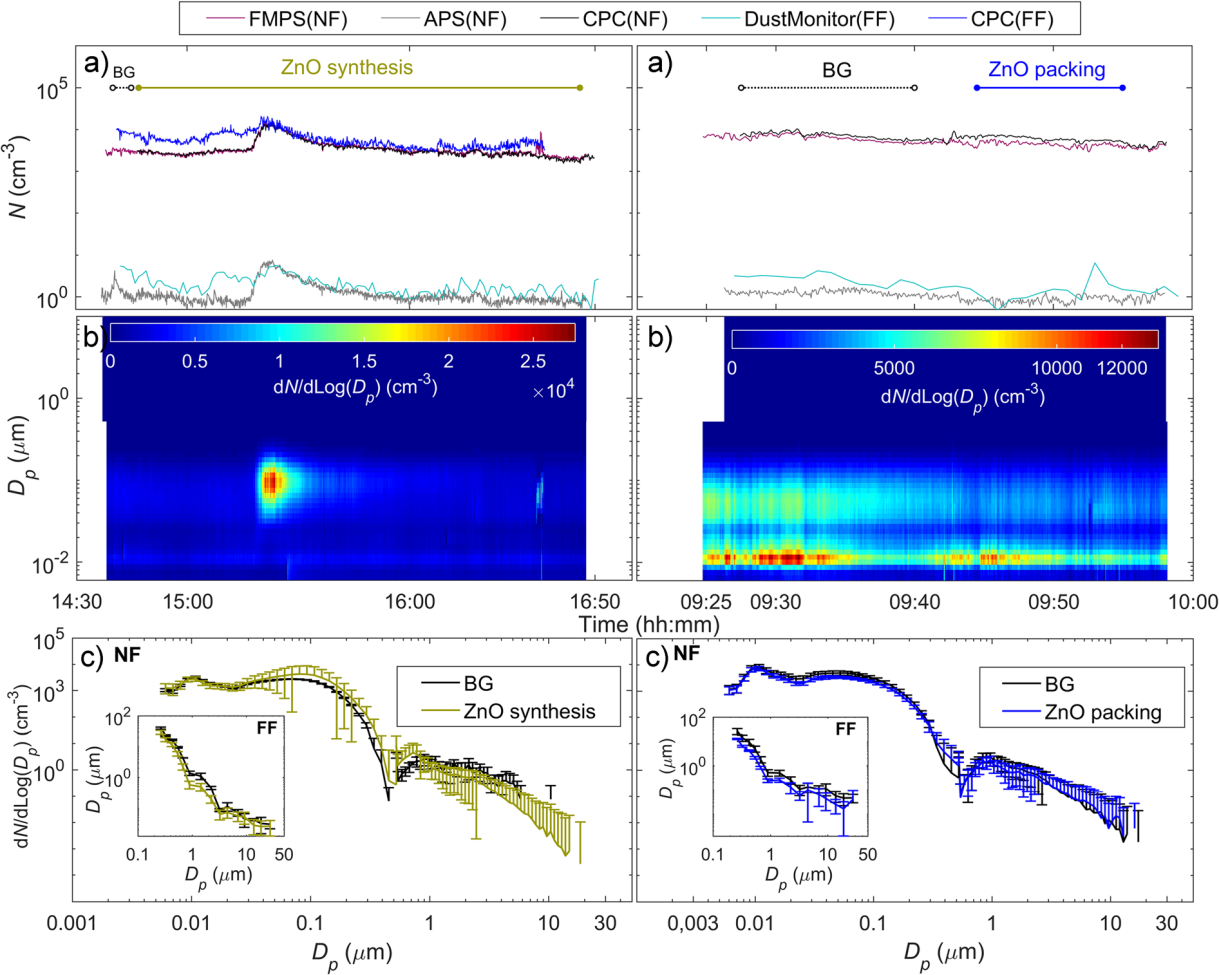
Time series of **a** particle number concentrations measured simultaneously at NF and FF during ZnO synthesis (calcination, transferring, and natural cooling down of the produced ZnO) and packing; **b** particle number size distributions obtained by the combination of FMPS and APS (range 5.6 nm–20 μm) in the NF; and **c** mean particle size distribution measured by FMPS and APS in NF and by OPS in FF during each task. The whiskers show the standard deviation. (For interpretation of the references to colour in this figure legend, the reader is referred to the web version of this article)

Particle measurements in laboratory B covered synthesis, as well as handling and packaging activities of ZnO and TiO_2_ nanoparticles (Fig. 5; Fig. S1 and Table S3 in the Electronic supplementary material). The real-time measurements showed nearly constant particle number concentrations and similar particle size distributions in both the NF and FF during BG and packing of ZnO and TiO_2_. The NF and FF CPC measurements showed minor deviations from the trends measured by the other instruments, which is ascribed to particles in the smallest nanosize ranges that can be detected by the CPC. However, an increased particle concentration (> 2 × 10^4^ cm^−3^) was observed for a short-term period of approximately 10 min in both the NF and FF during synthesis of TiO_2_ and ZnO (calcination process). Figure 5b and Fig. S1b revealed that the concentration of particles < 10 nm and > 1 μm were not affected by this particular incident. A comparison between the mean particle number size distribution observed prior to and at the moment of this concentration peak is shown in Fig. S2. This incidental episode was linked to outdoor particle sources, from smoking cigarettes that occurred in neighbouring environments.

The personal and stationary respirable dust concentration levels were again below the minimum detection limits in both cases of synthesis and handling ZnO and TiO_2_.

### Simulated spill test

**Fig. 6:**
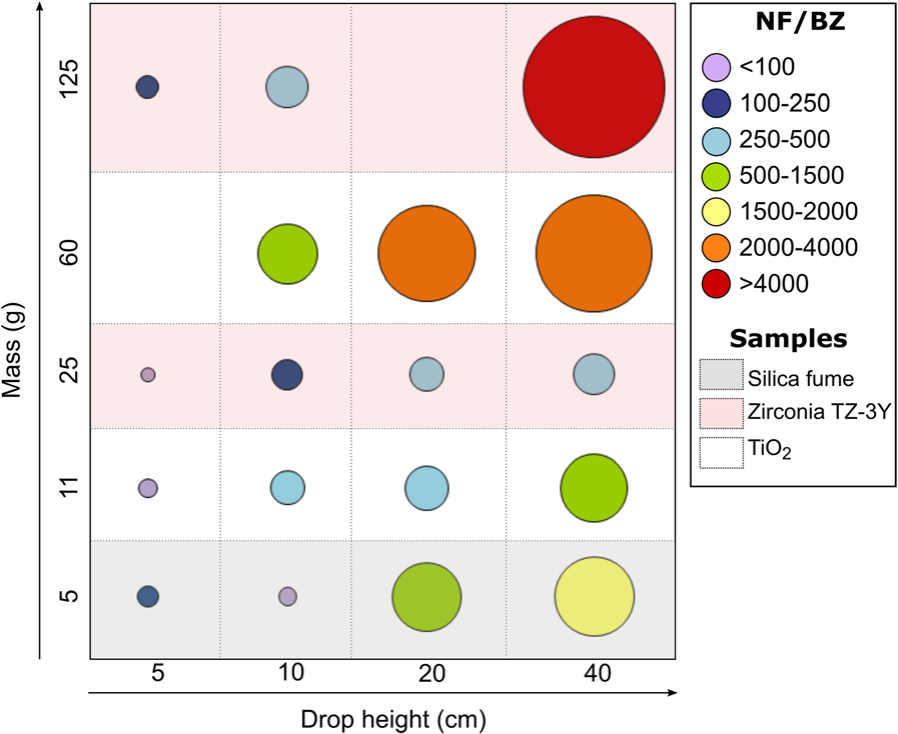
Bubble plot of the ratios NF/BZ obtained during drop tests as a function of drop height and mass loads. Note: The area of each circle is proportionate to the ratio NF/BZ

Figure 6 and Table 2 show the NF/BZ concentration ratios (BG corrected) calculated from the total particle number concentrations (see Table S4 of the Electronic supplementary material). These ratios varied from 1.4 to 1.3 × 10^4^, and it was found that the NF concentration levels depends on the amount of NM spilled and the drop height—the higher the mass load and drop height, the higher the NF/BZ ratio (Fig. 6). The largest amount of spilled NM from 40 cm (125 g of zirconia TZ-3Y) was five times greater than the smallest amount (25 g of zirconia TZ-3Y), and the ratio of the released particles in NF in terms of particle number concentration was greater than a factor of 3.3 (Table S4). At the same drop height, a factor of 1.4 was found for 60 g TiO_2_ when compared with the mass load of 11 g.

**Table 2:** Background corrected particle number concentrations ratios NF/BZ and fume hood protection factors during drop tests

Material	Mass (g)	Drop height							
		5 cm		10 cm		20 cm		40 cm	
		NF/BZ × 10^2^	*ε* (%)	NF/BZ × 10^2^	*ε* (%)	NF/BZ × 10^2^	*ε* (%)	NF/BZ × 10^2^	*ε* (%)
Silica fume	5	1.2	99.2	0.8	98.7	11.8	99.9	15.7	99.9
Zirconia TZ-3Y	25	0.01	77.8	2.3	99.6	2.8	99.8	4.2	99.7
	125	1.2	99.2	4.3	99.7	N/A	N/A	128.7	99.9
TiO_2_	11	0.9	98.9	2.8	99.8	4.8	99.8	11.2	99.9
	60	N/A	N/A	8.8	99.9	23.1	99.9	33.6	99.9

N/A, not available data

**Fig. 7:**
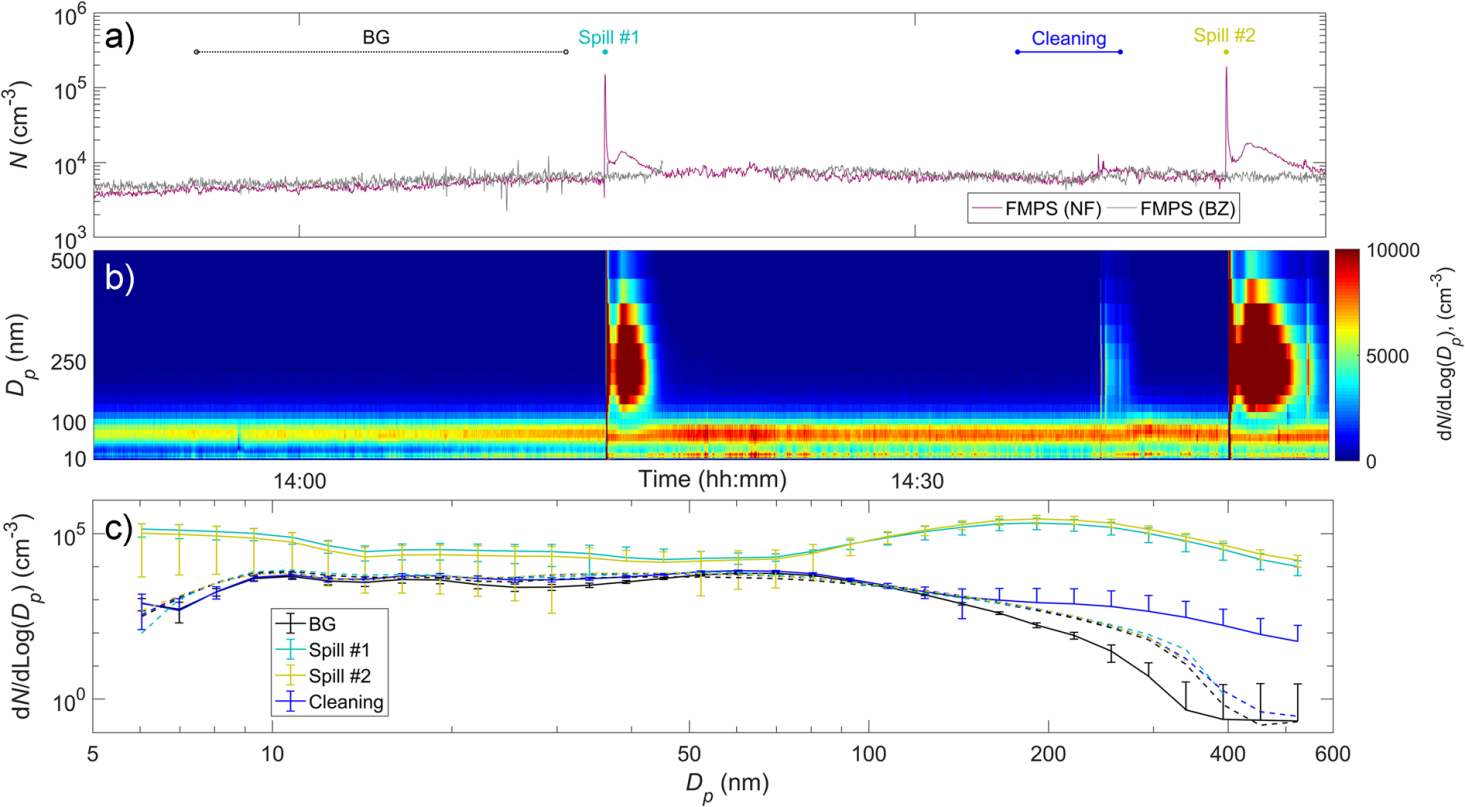
**a** Particle number concentration measured with FMPS during spillage of 60 g of ultrafine TiO_2_; **b** particle number size distribution measures with FMPS in NF; and **c** mean particle number size distribution measured with FMPS in NF (solid lines) and BZ (dashed lines) before the spillage, during the NM drops and during cleaning activity

Figure 7a, b illustrate the time series of the total particle number concentrations and the size distribution during the highest dustiness index material drop test (60 g TiO_2_ from 40 cm drop height). Two major peaks at the instances of drops (two replicas) were detected inside the fume hood (NF) by reaching 1.5 × 10^5^ cm^−3^ and 1.9 × 10^5^ cm^−3^, respectively. However, this did not result in significant increase of particle number concentration in the worker’s BZ (Fig. 1b). The BZ particle levels during drop tests were in the same order of magnitude as in the BG (7 × 10^3^ cm^−3^; Table S4).

All particle size distributions measured in NF during the NM spillage showed bimodal curves with one peak at nanosize range (< 10 nm) and another at 200 nm which can be interpreted as agglomerated TiO_2_ particles (Fig. 7b, c). The particle number concentration size distributions in the NF followed a similar pattern as during non-activity periods, except for > 200-nm-size particles where higher particle number concentration was detected (Fig. 7b, c). In contrast, the simultaneous size distributions measured in the worker BZ showed consistently lower multimodal curves similarly to BG (differences < 15%). The cleaning of the contaminated surface was the main task performed between drop tests. Once again, no significant increase in particle concentrations was detected in the BZ during cleaning of spilled particles and thus, particle size distributions were nearly the same as BG.

The fume hood protection factors shown in Table 2 indicate a pronounced mean efficacy of 98.3% and suggest that fume hood effectiveness is independent of the type NM.

Even though the fume hoods appeared to protect well against airborne particle exposure during accidents, care must still be taken. In some cases, such as for a drop test using 25 g zirconia TZ-3Y (drop height of 5 cm), powder was observed to eject from the fume hood and contaminated the technician, work chair and floors (see Fig. S3 of the Supplementary information). In this accidental situation, a smaller number of airborne particles did escape into the laboratory air but were barely detected by the particle monitors (in the range of 5.6–560 nm). Such incidents can lead to the lower fume hood protection factors (78%) occasionally observed. In such cases, care must be taken to apply proper spill cleaning procedures and use of adequate exposure protection (NIOSH 2012). This type of spills may occur at different scales and be even not visible.

## Discussion

This study has shown no significant increase in the particle number concentration measured in NF, directly at the side of the worker during handling and packaging activities of ZnO and TiO_2_ nanoparticles (Table S3). Particle number concentrations and size distributions measured both in NF and FF remained nearly constant and close to BG levels (ranging from 2.4 × 10^3^ to 6.1 × 10^3^ cm^−3^). Similar results were observed by Plitzko (2009) who showed that a fume hood prevented nanoparticle release to the laboratory room during handling of synthetic ceramic nanoparticles and nanofibers.

The particle concentrations during synthesis and handling of CuO nanoparticles were highest during milling (NF/BZ = 11.7), cooling CuO (NF/BZ = 3.7) and transferring activities (NF/BZ = 2.8); whereas, the ratio NF/BZ was nearly unit during the calcination process (Table S2). These increased concentrations were dominated by particles in the nanosize range (< 58 nm; Figs. 3 and 4) suggesting that NM exposure may occur if the fume hood is not working properly. However, assuming a hypothetical scenario where CuO nanoparticles escaped into the workplace, the exposure concentration levels (assumed to be the same as in NF; Table S2) would not exceed the short-term 15 min time-weighted average nanoreference value of 8 × 10^4^ cm^−3^ (NRV_15-min TWA_ established by the Social and Economic Council of the Netherlands for particles with density < 6 × 10^3^ kg m^−3^; SER 2012) at none of the activities involved in CuO handling and synthesis.

In order to increase confidence in worker protection by fume hoods, we challenged a standard laboratory fume hood and studied its efficacy by simulating spillage using different NMs, drop heights and mass loads. The drop tests considered in this study confirmed that the higher the mass load and drop height, the higher the nanoparticle emissions under the fume hood. The NF particle number concentration was up to 8.4 × 10^5^ cm^−3^ during a spillage of 125 g zirconia TZ-3Y at the highest drop height (40 cm); whereas, at the lowest drop height (5 cm), the concentrations were 2 orders of magnitude lower (maximum of 6.3 × 10^3^ cm^−3^). Same conclusion can be drawn when dealing with larger amounts of NMs: five times larger amounts of zirconia TZ-3Y (125 g vs. 25 g), lead to a greater total particle number concentration by a factor of 3.3 (Table S4). Previous findings seem to be in agreement with Tsai et al. (2009) who noticed that handling of 100 g nanoalumina results in greater extent of particle release than did smaller amount of 15 g nanoalumina (by a factor of 6). As for the type of spilled NM (corresponding to different dustiness indices and different number of particles generated during the single drop and the rotation test), no clear results were obtained (Fig. 6; Table 2). The reason of higher nanoparticle emissions detected inside the fume hood during spillage of zirconia TZ-3Y (DI_Inhalable_ = 283 ± 43 mg kg^−1^; *S*_single drop_ = 0.06 × 10^−7^ μm^3^ s^−1^; and *S*_continuous rotation_ = 4.8 × 10^−7^ μm^3^ s^−1^) than a powder with higher inhalable dustiness level and total respirable volume emission during the single drop and the rotation test (TiO_2_; DI_Inhalable_ = 8338 ± 233 mg kg^−1^; *S*_single drop_ = 17.2 × 10^−7^ μm^3^ s^−1^; and *S*_continuous rotation_ = 264.3 × 10^−7^ μm^3^ s^−1^) is unknown. However, the drop tests considered in this study did not result in any significant particle release from the fume hood to reach the worker’s BZ. The BZ particle levels during drop tests were at the same order of magnitude as in the BG (7 × 10^3^ cm^−3^; Table S4).

Results from this study seem to be inconsistent with Tsai et al. (2009) and Lee et al. (2011) who found that the handling of 15 g nanoalumina and nanosilver (Tsai et al. 2009) or nano-TiO_2_ manufacturing (Lee et al. 2011) in fume hoods can result in a significant release of airborne nanoparticles into the laboratory environment and the researcher’s breathing zone.

In overall, this study confirms that properly used standard fume hoods prevent well against particle release into the general laboratory environment. The average in-use protection efficacy was 98.3% with a total range from 78 to 99%. The obtained efficacy values were in the same range or even greater than the not strictly nanospecific values found in the exposure control efficiency library (ECEL; Fransman et al. 2008), specifically for local exhaust ventilation systems with an additional encapsulation or encasing of the source (95% confidence interval = 69–94%).

## Conclusions

In this research, the potential release and the workers’ inhalation exposure associated with the synthesis and handling of CuO, ZnO and TiO_2_ under a laboratory fume hood were assessed. In addition, the capacity of a fume hood to prevent particle release to laboratory air during simulated spillage of three different NMs (silica fume, zirconia TZ-3Y and TiO_2_) by varying drop height and mass load was evaluated. Airborne particle concentrations (2.5 nm–20 μm size ranges) were measured simultaneously in near-field, far-field and breathing zones of the worker using real-time particle counters. Samples were also collected for gravimetric, microscopy and chemical analysis. The main findings are summarised as follows:

- Milling, transferring and cooling CuO nanoparticles inside the fume hood-generated particles with significantly low particle diameters (< 58 nm) in terms of particle number concentration (up to 1 × 10^5^ cm^−3^).
- Measurements conducted in near field, directly at the side of the worker (< 1 m from fume hood opening) during synthesis, handling and packaging activities of ZnO and TiO_2_ nanoparticles, did not result in significant increase of particle number concentration compared with far-field concentrations. Particle number concentrations measured both in near field and far field remained nearly constant (~ 1 × 10^3^ cm^−3^).
- Simulated powder spills showed a systematic increase in the particle concentrations inside the fume hood with increasing mass load and drop height but did not result in NMs being released into the general laboratory environment. Despite powder spills were sometimes observed to eject into the laboratory room and contaminate the workers’ laboratory clothing, the spill events were rarely associated with notable release of particles (in the range of 5.6–560 nm) from the fume hood.
- The fume hood protection factors indicated a mean efficacy of 98.3% with a total range from 78 to 99% and suggested that fume hood effectiveness is independent of the type NM.

In overall, this study confirms that an appropriate fume hood with an adequate sash height of 0.3–0.5 m and face velocities ranging from 0.1 to 0.4 m s^−1^ is sufficient exposure control during sol-gel synthesis and handling of NMs. Nevertheless, the standard approached for cleaning powder spills should be used to prevent exposure via resuspension and inadvertent exposure by secondary routes. Furthermore, a regularly fume hood’s operational status checking is recommended.

## Electronic supplementary material

ESM 1 (DOCX 3555 kb)
