# Supplementary material for: Particle release and control of worker exposure during laboratory-scale synthesis, handling and simulated spills of manufactured nanomaterials in fume hoods
Source: J Nanopart Res. 2018 Feb 21;20(2):48. doi: 10.1007/s11051-018-4136-3 (PMC5820406; doi:10.1007/s11051-018-4136-3)
Supplement: Supplementary file 1 — (DOCX 3555 kb) [file 11051_2018_4136_MOESM1_ESM.docx]

**Supplementary information**

**Particle release and control of worker exposure during laboratory-scale synthesis, handling and simulated spills of manufactured nanomaterials in fume-hoods**

Ana S. Fonseca^1,^*, Eelco Kuijpers^2^, Kirsten I. Kling^1^, Marcus Levin^1^, Antti J. Koivisto^1^, Signe H. Nielsen^1^, W. Fransman^2^, Yijri Fedutik^3^,, Keld A. Jensen^1^, Ismo K. Koponen^1^

^1^ National Research Centre for the Working Environment (NRCWE), Lerso Parkallé 105 DK-2100 Copenhagen, Denmark

^2^ TNO, Risk Analysis for Products in Development, Zeist, The Netherlands

^3^ PlasmaChem GmbH, Schwarzschildstr 10, 12489 Berlin, Germany

*Author to whom correspondence should be addressed: A. S. Fonseca ([agf@nrcwe.dk](mailto:agf@nrcwe.dk)); Phone: +45 39 16 54 92

***Table S1*** *Physicochemical characteristics and morphology of the pristine inorganic nanostructured NM under study (data provided by the manufacturer or referenced if otherwise).*

| Sample | *DI_resp_* ^a^  (mg kg^-1^) | Primary size  (nm) | Specific surface area  (m^2^ g^-1^) | Bulk density  (g cm^-3^) | Surface elemental composition ^b^ | Morphology by  TEM/SEM analysis |
| --- | --- | --- | --- | --- | --- | --- |
| CuO  (CAS No.1317-38-0) | 104 | 40±10 | 15±5 | 0.8 | N/A | 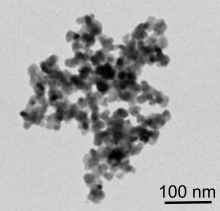 |
| ZnO  (CAS No.1314-13-2) | 258.8±76.1 | 13.2±5.4 | 30±5 | 5.61 | 16.9 % C, 50.9 % O, 32.2 % Zn | 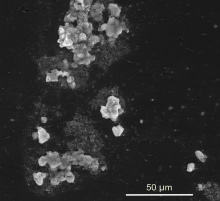 |
| TiO_2_ anatase  HNO_3_ stabilised  (CAS No.13463-67-7) | 1.5±2.2* | 1-10 | 140 | 4.23 | 21.5 % C, 42.2 % O, 36.3 % Ti | 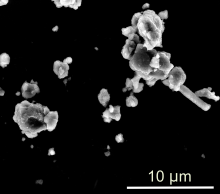 |

^a^ Dustiness tests were conducted using the EN15051 continuous drop method (Jensen et al. 2016) for CuO and the downscaled rotating dustiness drum for both ZnO and TiO_2_ (Jensen et al. 2009; Levin et al. 2015)

^b^ Analysed by X-ray photoelectron spectroscopy (XPS) (Levin et al. 2015)

* The $\surd\sigma$ replaced the average, which was below the detection limit of quantification; The $\sigma$ corresponds to the standard deviation.

N/A : Not available data

***Table S2*** *Descriptive statistics for the measured particle number concentrations (mean, standard deviation σ and ratios NF/FF) under laboratory work during synthesis and handling CuO.*

|  |  | ***Instruments*** | | | | | | | | |
| --- | --- | --- | --- | --- | --- | --- | --- | --- | --- | --- |
|  |  | **DiSCmini** | | | **OPS** | | | **CPC^b^** | | |
| **Activity** | **Location^a^** | Mean *N* ×10^2^ | *± σ* ×10^2^ | Ratio | Mean *N* ×10^2^ | *± σ* ×10^2^ | Ratio | Mean *N* ×10^2^ | *± σ* ×10^2^ | Ratio |
|  |  | (cm^-3^) | (cm^-3^) | NF/FF | (cm^-3^) | (cm^-3^) | NF/FF | (cm^-3^) | (cm^-3^) | NF/FF |
| **BG** | NF | 16.7 | 4.4 | 1.3 | 0.34 | 0.08 | 1 | N/A | N/A | N/A |
|  | FF | 12.7 | 5.4 |  | 0.34 | 0.02 |  | 4.85 | 1.22 |  |
| **Milling (M)** | NF | 116.2 | 185.2 | 11.7 | 0.72 | 0.15 | 2.4 | N/A | N/A | N/A |
|  | FF | 9.9 | 3.2 |  | 0.3 | 0.01 |  | 4.84 | 0.26 |  |
| **Transferring (T)** | NF | 26.8 | 4.6 | 2.8 | 0.74 | 0.16 | 2.4 | N/A | N/A | N/A |
|  | FF | 9.5 | 1.0 |  | 0.3 | 0.01 |  | 4.78 | 0.42 |  |
| **Calcination** | NF | 19.6 | 3.7 | 1.2 | 0.39 | 0.11 | 1.2 | N/A | N/A | N/A |
|  | FF | 16.5 | 4.6 |  | 0.31 | 0.03 |  | 7.33 | 1.53 |  |
| **Cooling CuO** | NF | 55.6 | 158.1 | 3.7 | 0.37 | 0.07 | 1.3 | N/A | N/A | N/A |
|  | FF | 15.1 | 1.9 |  | 0.29 | 0.01 |  | 6.98 | 0.64 |  |

^a^ NF: inside the fume-hood (in cm range from the emission source); FF: 4 m away from fume-hood

^b^ CPC, TSI model 3007 used both in FF

N/A : Not available data

***Table S3*** *Descriptive statistics for the measured particle number concentrations (mean, standard deviation σ and ratios NF/FF) under laboratory work during synthesis and handling ZnO and TiO_2_.*

|  |  |  | ***Instruments*** | | | | | | | | | | | |
| --- | --- | --- | --- | --- | --- | --- | --- | --- | --- | --- | --- | --- | --- | --- |
| **Activity** | | **Location^a^** | **CPC^b^** | | | **FMPS** | | | **APS** | | | **DustMonitor** | | |
| ***Real case scenario: ZnO*** | | | Mean *N*  ×10^2^ | *± σ*  ×10^2^ | Ratio | Mean *N*  ×10^2^ | *± σ*  ×10^2^ | Ratio | Mean *N* | *± σ* | Ratio | Mean *N* | *± σ* | Ratio |
|  |  |  | (cm^-3^) | (cm^-3^) | NF/FF | (cm^-3^) | (cm^-3^) | NF/FF | (cm^-3^) | (cm^-3^) | NF/FF | (cm^-3^) | (cm^-3^) | NF/FF |
| BG | | NF | 30.8 | 0.7 | 0.4 | 30.3 | 1.1 | N/A | 2.02 | 0.42 | N/A | N/A | N/A | N/A |
|  |  | FF | 72.8 | 4.4 |  | N/A | N/A |  | N/A | N/A |  | 1.95 | 0.03 |  |
| ZnO synthesis | | NF | 36.8 | 22 | 0.6 | 38.6 | 24.1 | N/A | 3.28 | 3.49 | N/A | N/A | N/A | N/A |
|  |  | FF | 60.5 | 28.9 |  | N/A | N/A |  | N/A | N/A |  | 3.39 | 3.23 |  |
| Packing | | NF | 60.5 | 7.8 | N/A | 44.5 | 6 | N/A | 1.4 | 0.36 | N/A | N/A | N/A | N/A |
|  |  | FF | N/A | N/A |  | N/A | N/A |  | N/A | N/A |  | 0.91 | 0.08 |  |
| ***Real case scenario: TiO_2_*** | | | Mean *N*  ×10^2^ | *± σ*  ×10^2^ | Ratio | Mean *N*  ×10^2^ | *± σ*  ×10^2^ | Ratio | Mean *N* | *± σ* | Ratio | Mean *N* | *± σ* | Ratio |
|  |  |  | (cm^-3^) | (cm^-3^) | NF/FF | (cm^-3^) | (cm^-3^) | NF/FF | (cm^-3^) | (cm^-3^) | NF/FF | (cm^-3^) | (cm^-3^) | NF/FF |
| BG | | NF | 24.2 | 0.9 | 0.8 | 25.5 | 2 | N/A | 2.98 | 0.51 | N/A | N/A | N/A | N/A |
|  |  | FF | 29.8 | 13.4 |  | N/A | N/A |  | N/A | N/A |  | 2.71 | 0.44 |  |
| TiO_2_ synthesis | | NF | 46 | 55.6 | 0.9 | 49.6 | 65.6 | N/A | 2.49 | 0.99 | N/A | N/A | N/A | N/A |
|  |  | FF | 48.4 | 60.2 |  | N/A | N/A |  | N/A | N/A |  | 1.22 | 0.63 |  |
| Packing | | NF | 35.3 | 2.2 | 0.9 | 35 | 2.5 | N/A | 3.62 | 0.55 | N/A | N/A | N/A | N/A |
|  |  | FF | 39.8 | 12.1 |  | N/A | N/A |  | N/A | N/A |  | 1.59 | 0.07 |  |

^a^ NF: directly at the side of the worker <1 m from fume-hood; FF: 4 m away from fume-hood

^b^ CPC, TSI model 3007 used in NF and UWCPC, TSI model 3786 used in FF

N/A : Not available data

***Table S4*** *Measured particle number concentrations (N, cm^-3^) during drop tests. In parenthesis, the ±σ corresponds to the standard deviation.*

|  |  |  | **Drop height (cm)** | | | |  |
| --- | --- | --- | --- | --- | --- | --- | --- |
| **Material** | **Mass (g)** | **Activity/location** | | 5 | 10 | 20 | 40 |
|  |  |  |  | *N* ×10^3^ (*± σ* ×10^3^) | | | |
|  |  |  |  | (cm^-3^) | | | |
| Silica fume | 5 | BG | NF | 1.8 (0.7) | 4.5 (0.7) | 3.6 (0.3) | 3.8 (0.5) |
|  |  |  | BZ | 2.0 (0.7) | 5.1 (0.2) | 4.6 (0.4) | 4.8 (0.5) |
|  |  | Spill | NF | 4.7 (2.0) | 11 (7.7) | 9.9 (5.5) | 42 (28) |
|  |  |  | BZ | 2.0 (0.2) | 5.2 (0.3) | 4.6 (0.3) | 4.8 (0.4) |
| Zirconia TZ-3Y | 25 | BG | NF | 1.3 (0.2) | 1.6 (0.2) | 1.2 (0.2) | 1.6 (0.4) |
|  |  |  | BZ | 1.5 (0.5) | 3.3 (0.5) | 2.2 (0.3) | 2.0 (0.2) |
|  |  | Spill | NF | 2.6 (1.2) | 33 (31) | 123 (124) | 91 (116) |
|  |  |  | BZ | 2.4 (1.2) | 3.4 (0.4) | 2.2 (0.2) | 2.2 (1.5) |
|  | 125 | BG | NF | 1.0 (0.2) | 1.0 (0.2) | N/A | 1.3 (0.2) |
|  |  |  | BZ | 0.8 (0.1) | 0.7 (0.1) | N/A | 1.1 (0.5) |
|  |  | Spill | NF | 3.8 (2.2) | 76 (40) | N/A | 300 (328) |
|  |  |  | BZ | 0.8 (0.02) | 0.9 (0.2) | N/A | 1.2 (0.3) |
| TiO_2_ | 11 | BG | NF | 1.7 (0.2) | 1.6 (0.1) | 1.7 (0.4) | 2.4 (0.5) |
|  |  |  | BZ | 1.7 (0.4) | 4.3 (0.4) | 2.6 (0.9) | 4.6 (1.2) |
|  |  | Spill | NF | 38 (10) | 25 (20) | 196 (155) | 128 (121) |
|  |  |  | BZ | 2.2 (0.05) | 4.4 (0.5) | 2.7 (0.6) | 4.7 (0.8) |
|  | 60 | BG | NF | N/A | 3.0 (0.4) | 4.9 (0.8) | 4.3 (0.4) |
|  |  |  | BZ | N/A | 2.7 (0.4) | 6.0 (1.2) | 6.6 (0.5) |
|  |  | Spill | NF | N/A | 19 (14) | 396 (216) | 175 (23) |
|  |  |  | BZ | N/A | 2.7 (0.2) | 6.2 (0.7) | 6.7 (0.4) |

N/A : Not available data


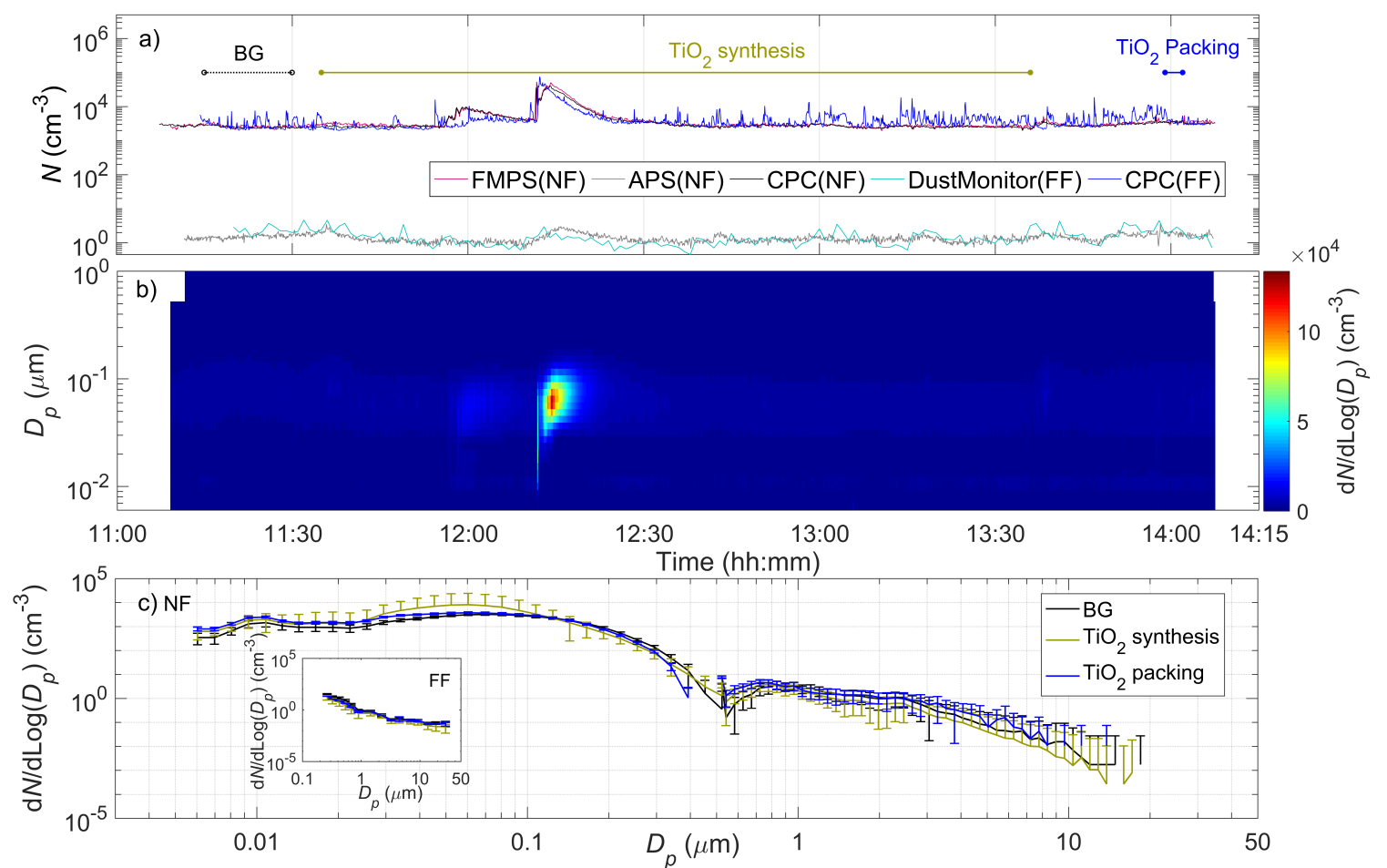


***Fig.S1*** *Time series of (a) particle number concentrations measured simultaneously at NF and FF during TiO_2_ synthesis (calcination, transferring and natural cooling down of the produced TiO_2_), and packing, (b) particle number size distributions obtained by the combination of FMPS and APS (range 5.6 nm-20 µm) in the NF and (c) mean particle size distribution measured by FMPS and APS in NF and by OPS in FF during each task. The whiskers show the standard deviation. (For interpretation of the references to colour in this figure legend, the reader is referred to the web version of this article)*


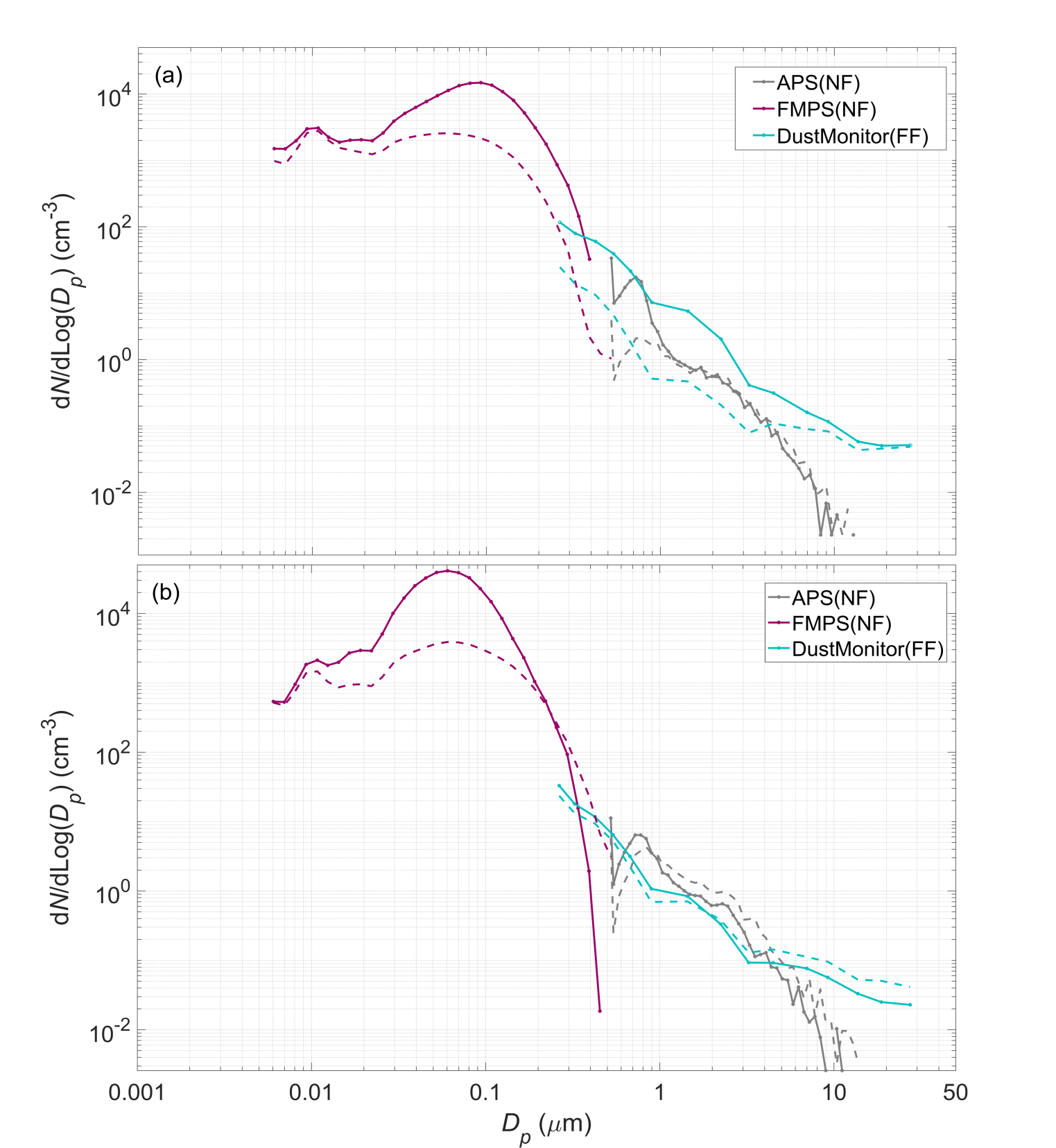


***Fig.S2*** *Particle number size distribution observed at the concentration peak (solid lines) during synthesis of (a) ZnO and (b) TiO_2_. Dashed lines correspond to the size distribution obtained during synthesis prior the detection of the peak in total number concentration*

*
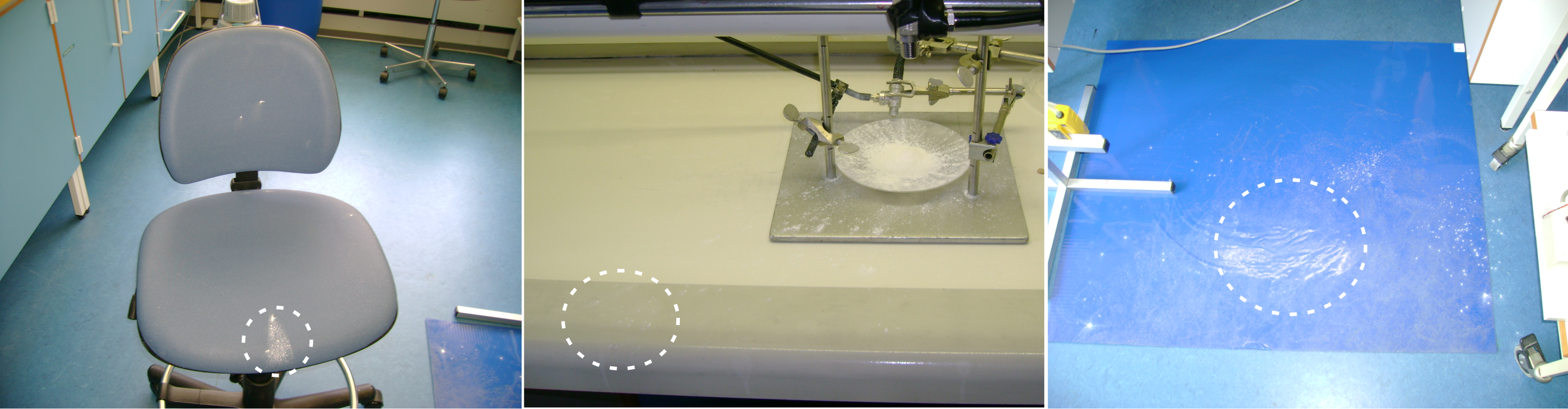
****Fig.S3*** *Powder splashes on the laboratory chair, edge of the fume-hood and on the floor in front of the fume-hood*
